# Molecular Lineage Replacement and Shifted Seasonality of Pediatric Respiratory Syncytial Virus on Tropical Hainan Island, China, 2021–2024

**DOI:** 10.3390/pathogens15020182

**Published:** 2026-02-06

**Authors:** Yibo Jia, Siqi Chen, Shannan Wu, Ruoyan Peng, Yi Huang, Gaoyu Wang, Meng Chang, Meifang Xiao, Yueqing Chen, Yujuan Guo, Feifei Yin

**Affiliations:** 1 Department of Medical Administration, Hainan General Hospital (Hainan Affiliated Hospital of Hainan Medical University), Haikou 570311, China; yibojia1@gmail.com; 2 The University of Hong Kong Joint Laboratory of Tropical Infectious Diseases, Key Laboratory of Tropical Translational Medicine of Ministry of Education, Hainan Medical University, Haikou 571199, China; qisi49u@muhn.edu.cn (S.C.);; 3 Animal and Plant Quarantine Center Haikou Customs District, Haikou 570311, China; 4 Department of Clinical Laboratory, Center for Laboratory Medicine, Hainan Women and Children’s Medical Center, Hainan Medical University, Haikou 570206, China; 5 Department of Hematology, Hainan General Hospital (Hainan Affiliated Hospital of Hainan Medical University), Haikou 570311, China

**Keywords:** respiratory syncytial virus (RSV), pediatric acute respiratory tract infections, seasonality shift, non-pharmaceutical interventions (NPIs), post–Zero-COVID period, molecular epidemiology, lineage replacement, G glycoprotein glycosylation

## Abstract

Respiratory syncytial virus (RSV) resurged in many regions after the relaxation of stringent non-pharmaceutical interventions (NPIs) implemented during the COVID-19 pandemic. Here, we characterized the epidemiological patterns and molecular evolution of RSV among pediatric inpatients with acute respiratory tract infections (ARTIs) on tropical Hainan Island, China. We retrospectively analyzed 32,329 children (≤18 years) hospitalized at Hainan Women and Children’s Medical Center from January 2021 to December 2024. RSV positivity was determined using targeted next-generation sequencing. In total, 4483/32,329 (13.86%) patients were RSV-positive, with a high positivity in 2021 (20.27%, 957/4721), marked suppression in 2022 (2.03%, 106/5227) during intensive NPIs, and a rebound in 2023–2024 (15.31%, 1490/9732; 15.26%, 1930/12,649). RSV positivity was higher in boys than girls (14.42% vs. 13.00%). Seasonality shifted from a summer–autumn peak in 2021 to a spring–summer predominance in 2023–2024. Among 56 sequenced RSV-positive specimens (29 RSV-A; 27 RSV-B), all RSV-A strains belonged to genotype ON1 (lineages A.D.3 and A.D.5.2), and all RSV-B strains belonged to genotype BA9 (lineages B.D.4.1.1, B.D.E.1, and B.D.E.2). Subtype dominance transitioned from RSV-A (2021–2023; mainly A.D.3) to RSV-B in 2024 (all B.D.E.1). Lineage-specific amino-acid and predicted N-glycosylation changes were observed, including loss of the N179 site in A.D.5.2 and acquisition of N258 in B.D.E.1. These findings indicate that RSV circulation on tropical Hainan was strongly suppressed during intensive NPIs and re-established after policy relaxation, accompanied by earlier seasonal activity and clear lineage replacement, underscoring the need for sustained genomic surveillance to inform locally tailored clinical preparedness and immunization strategies.

## 1. Introduction

Human respiratory syncytial virus (RSV) is the most commonly identified viral pathogen in pediatric acute lower respiratory infections (ALRIs), with an estimated annual incidence exceeding 33 million cases worldwide, resulting in over 3 million hospitalizations and nearly 100,000 deaths in children under five [1]. Despite the recent availability of long-acting monoclonal antibodies and maternal vaccines, RSV continues to impose a heavy burden on health-care systems, particularly in low- and middle-income countries where access to preventive interventions remains limited [2].

RSV is classified into two major antigenic and genetic groups, RSV-A and RSV-B, based on variability in the attachment (G) glycoprotein gene [3]. Within each group, multiple genotypes and lineages have been recognized, many of which are characterised by a partial duplication in the second hypervariable region of the G gene—designated ON1 for RSV-A and BA for RSV-B [4]. These duplications, together with extensive O- and N-linked glycosylation, shape the antigenic surface of the G protein and may facilitate immune evasion. Structural studies have shown that antibodies targeting the central conserved region of G can mediate broad neutralisation, underscoring the need for continued molecular surveillance of circulating strains.

Before the COVID-19 pandemic, RSV epidemics in temperate regions typically peaked in winter. In contrast, in tropical and subtropical areas RSV usually circulated year-round with one or two broad peaks, often coinciding with the rainy or humid season rather than a distinct winter period. In many tropical settings, including southern China and Southeast Asia, RSV activity increases during warm, rainy months and exhibits less pronounced off-season troughs than in temperate climates. These geographical differences in seasonality are critical for the design and timing of immunoprophylaxis and other RSV control strategies.

The COVID-19 pandemic and associated non-pharmaceutical interventions (NPIs)—such as masking, school closures, travel restrictions and lockdowns—profoundly altered the circulation of seasonal respiratory viruses. Many countries reported an almost complete disappearance of RSV and influenza in 2020–2021, followed by atypical, often off-season resurgences after relaxation of NPIs [5,6,7,8,9]. In China, stringent containment policies and the Dynamic Zero-COVID strategy were maintained until 7 December 2022, when nationwide restrictions were abruptly lifted [10]. Subsequent reports from Beijing and other regions described intense RSV epidemics with altered seasonality and age distribution, as well as the emergence of novel lineages such as A.D.5.2 and B.D.E.1 that contributed to large post–Zero-COVID RSV waves [11]. However, most available data originate from temperate, highly urbanised settings, and the impact of NPIs and policy changes on RSV dynamics in tropical environments remains poorly defined.

Hainan Island, located at the southernmost tip of China, is the country’s only tropical island province. Its warm, humid climate, distinct monsoon pattern and role as a free-trade-port tourism hub create unique conditions for respiratory virus transmission. Hainan experienced relatively limited but clearly demarcated waves of COVID-19, accompanied by stringent, time-bound NPIs and a synchronised withdrawal of the Dynamic Zero-COVID policy with the rest of mainland China. This combination of tropical climate, geographic semi-isolation and well-documented policy shifts provides a natural setting in which to examine how NPIs and their relaxation influenced RSV transmission dynamics and viral evolution in a paediatric population. Our previous work on influenza virus and rhinovirus among children on Hainan has shown that both viruses exhibited altered seasonal patterns and genetic diversity before and after the Dynamic Zero-COVID period, yet comparable data for RSV are lacking despite its central role in paediatric respiratory disease [12,13,14].

In this study, we conducted a retrospective analysis of paediatric inpatients with acute respiratory tract infections (ARTIs) at Hainan Women and Children’s Medical Center from January 2021 to December 2024. Using targeted next-generation sequencing (tNGS) for RSV detection and G-gene sequencing for lineage, phylogenetic and amino-acid analyses, we aimed to: (i) describe temporal trends and age distribution of RSV infection before and after the end of the Dynamic Zero-COVID policy; (ii) characterise shifts in RSV seasonality and subtype predominance; and (iii) investigate molecular lineage turnover and lineage-specific glycosylation patterns of circulating RSV-A and RSV-B strains on tropical Hainan Island. By integrating epidemiological and genomic data across a period of intensive NPIs and their withdrawal, this study seeks to inform local RSV prevention and clinical management and to enhance understanding of how major public-health interventions reshape RSV transmission and evolution in tropical settings.

## 2. Materials and Methods

### 2.1. Sample Collection and RSV Detection

This hospital-based retrospective surveillance included 32,329 pediatric inpatients (≤18 years old) with ARTIs admitted to Hainan Women and Children’s Medical Center between January 2021 and December 2024. Eligible children presented at admission with clinical manifestations consistent with ARTIs, such as fever, headache, rhinorrhoea, tachypnoea, cough, sore throat, sputum production, chest pain, or radiological evidence of pneumonia or bronchitis. Nasopharyngeal swabs were obtained from each patient on admission according to standard clinical procedures and were immediately placed in viral transport medium. Specimens were stored at −80 °C until further analysis. RSV infection was detected using a commercial targeted next-generation sequencing (tNGS) respiratory panel (KingMed Diagnostics, Guangzhou, China) as part of routine clinical testing. During 2021–2024, tNGS respiratory panel testing was routinely ordered at or near admission for pediatric ARTI inpatients at our center, and there were no hospital-wide changes in test-ordering policy, eligibility criteria or insurance-coverage restrictions. However, because testing was performed as part of real-world clinical care, clinician discretion in ordering cannot be fully excluded over time. In this study, RSV positivity is therefore reported as test positivity among tNGS-tested ARTI inpatients. The clinical report does not disclose internal panel versioning, sequencing instruments, or updates to the bioinformatics pipeline and reference databases. Thus, we did not claim a fixed panel “version” across years, but all specimens were processed by the same provider using a standardized clinical workflow and only QC-passed results were included. Sequencing data were processed through the provider’s standardized bioinformatics workflow and reporting framework, including quality control, alignment to reference databases, and read counting/normalization. The clinical report provides semi-quantitative metrics including normalized sequence counts (defined as organism-specific reads per 100,000 raw reads) and an estimated microbial load (copies/mL), and issues a final qualitative interpretation (positive/negative) according to validated QC and interpretation criteria; the analytical limit of detection is reported to be as low as 100 copies/mL. RSV positivity in this study was defined as RSV-A and/or RSV-B reported as positive (+) in the clinical report. The report does not disclose a single fixed minimal read depth/coverage cutoff for declaring positivity; therefore, we did not apply an additional user-defined read depth/coverage threshold beyond the laboratory’s qualitative call, and only QC-passed results (e.g., total reads, Q30, internal control, and positive/negative controls) were included. Co-detections of other respiratory pathogens in the panel were retained, and RSV-positive cases were counted as RSV-positive regardless of co-detection status. Samples included in downstream lineage analyses were further restricted to those yielding high-quality G-gene sequences. This study was approved by the Ethics Committee of Hainan Women and Children’s Medical Center (protocol code HNWCMC 2023 [111], approved on 9 November 2023).

### 2.2. RSV Classification Using G Gene Sequences

To characterize RSV lineages, 56 RSV-positive samples were selected for G-gene sequencing using a stratified, purposive sampling strategy to ensure coverage across different years (2021–2024) and epidemic phases (including periods of peak activity and low activity), while also aiming to include both RSV-A and RSV-B when available. When multiple eligible specimens were available within a given stratum, selection was guided by stronger sequencing signal on the clinical tNGS report to maximize amplification success. To assess representativeness, demographic and temporal characteristics (age, sex, and year of detection) were compared between the sequenced subset (*n* = 56) and all RSV-positive cases (*n* = 4483), as summarized in Table S1. Viral RNA was extracted from 200 μL of each specimen using the QIAamp Viral RNA Mini Kit (Qiagen, Valencia, CA, USA) according to the manufacturer’s protocol. Partial G-gene fragments encompassing the second hypervariable region were amplified by nested reverse-transcription PCR (RT-PCR) following the method described by Agoti et al. [15]. The first-round RT-PCR was performed using the SuperScript™ III One-Step RT-PCR System (Invitrogen, Carlsbad, CA, USA). Cycling conditions were 94 °C for 5 min; 40 cycles of 95 °C for 30 s, 55 °C for 30 s, and 72 °C for 5 min; and a final extension at 72 °C for 10 min. The second-round PCR was carried out with GoTaq™ Colorless Master Mix (Promega, Madison, WI, USA) using the same conditions except for an annealing temperature of 56 °C. A negative control was included in each run. PCR products from positive reactions were purified and subjected to Sanger sequencing on an ABI Prism 3730XL DNA Analyzer (Sangon Biotech, Guangzhou, China). Raw sequences were assembled and edited using SeqMan (DNASTAR, Madison, WI, USA), and viral identity was confirmed by BLAST (version 2.17.0) searches against the National Center for Biotechnology Information (NCBI) database.

### 2.3. Nucleotide Sequence Analysis

Nextclade (version 3.3.1; https://clades.nextstrain.org/ accessed on 4 March 2025) was used to assign RSV lineages and to identify amino-acid substitutions. All amino-acid changes were reported relative to the default reference sequences (RSV-A: hRSV/A/England/397/2017, EPI_ISL_412866; RSV-B: hRSV/B/Australia/VIC-RCH056/2019, EPI_ISL_1653999). Putative N-linked glycosylation sites were predicted using the NetNGlyc 1.0 server (http://www.cbs.dtu.dk/services/NetNGlyc/ accessed on 4 March 2025) with default settings. Because only HVR2-spanning partial G-gene fragments were generated in this study, glycosylation predictions were performed on the translated HVR2-containing G-protein fragments (N-X-S/T, X ≠ Pro) rather than the full-length G protein. Phylogenetic trees were constructed in MEGA X using the neighbor-joining method, with branch support evaluated by 1000 bootstrap replicates. Genetic diversity, pairwise sequence identity, and amino-acid variation were analyzed using BioEdit (version 7.1.3.0) software (https://thalljiscience.github.io/ accessed on 6 March 2025) [16]. All sequences generated in this study were deposited in GenBank under accession numbers OQ248592–OQ248607, OR140542–OR140554, and PV800154–PV800198.

### 2.4. Statistical Analysis

All statistical analyses were performed using IBM SPSS Statistics version 26.0 (IBM Corp., Armonk, NY, USA). Categorical variables were compared using the χ^2^ test. Two-tailed *p* values < 0.05 were considered statistically significant. Graphs were prepared using GraphPad Prism version 6.0 (GraphPad Software, San Diego, CA, USA).

## 3. Results

### 3.1. Sample Information and Demographics

From January 2021 to December 2024, 32,329 children with ARTIs were hospitalized at Hainan Women and Children’s Medical Center and included in the analysis. Patient ages ranged from 1 month to 18 years (mean ± SD, 2.97 ± 2.86 years). Boys accounted for 19,633 (60.73%) admissions and girls for 12,696 (39.27%), indicating a male predominance in the study population.

### 3.2. Analysis of RSV Infection Trends Before and After the Ending of Dynamic Zero-COVID Policy

**Figure 1:**
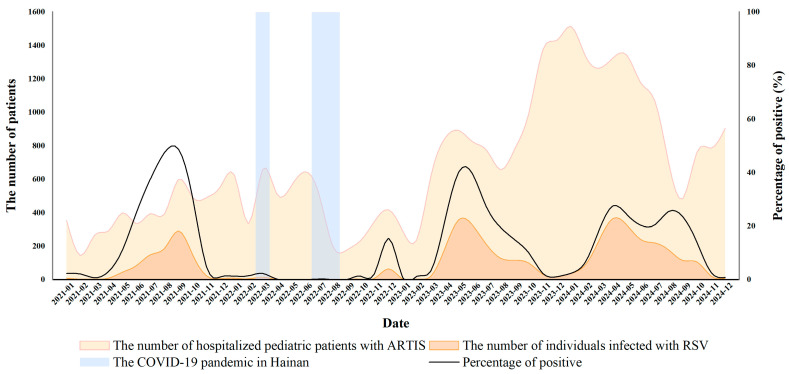
Monthly RSV activity among hospitalized pediatric patients with ARTIs in Hainan, January 2021–December 2024. Monthly numbers of hospitalized children with ARTIs (yellow shaded area), RSV-positive cases (orange shaded area), and RSV positivity (%) (black line, right y-axis). Blue shaded intervals indicate the periods of local COVID-19 outbreaks in Hainan. Abbreviations: RSV, respiratory syncytial virus; ARTI, acute respiratory tract infection.

Over the four-year period, 4483 of 32,329 pediatric inpatients with ARTIs tested positive for RSV, yielding an overall positivity rate of 13.86% (Table 1). These estimates reflect hospital-based test positivity among admitted ARTI cases rather than population-level RSV incidence. In 2021, 957 of 4721 ARTI inpatients were RSV-positive (20.27%). In 2022, although the number of ARTI hospitalizations increased slightly to 5227, only 106 patients were RSV-positive, and the annual positivity rate dropped sharply to 2.03%. This sharp decline coincided with two local COVID-19 outbreaks in March and August 2022 and the implementation of stringent non-pharmaceutical interventions. Following relaxation of the Dynamic Zero-COVID policy in December 2022, RSV circulation rebounded in 2023, with 1490 of 9732 ARTI inpatients testing RSV positive (15.31%). The infection rate remained high in 2024, with 1930 of 12,649 ARTI inpatients testing RSV positive (15.26%), reaching levels comparable to those observed in 2021 and indicating re-establishment of endemic transmission. The distribution of RSV positivity differed significantly by year (χ^2^ = 812.97, *p* < 0.001), indicating a profound temporal impact of COVID-19 control measures on RSV activity (Table 1, Figure 1).

RSV prevalence also differed by sex. Overall, the positivity rate among ARTIs inpatients was higher in boys than in girls (14.42% [2832/19,633] vs. 13.00% [1651/12,696]; χ^2^ = 13.03, *p* < 0.001). For comparison, the Seventh National Population Census reported a boys-to-girls ratio of 112.86 males per 100 females in Hainan in 2020. This male predominance persisted from 2022 to 2024, with significantly higher RSV positivity in boys than in girls in each year (Table 1).

Marked seasonal variation in RSV positivity was observed (Table 2). In 2021, detection rates rose from 5.85% in spring to 37.54% in summer, then declined to 29.20% in autumn and 1.61% in winter (χ^2^ = 636.76, *p* < 0.001). In 2022, RSV circulation was strongly suppressed throughout the year, with positivity rates of 0.93% in spring, 0.07% in summer, 1.57% in autumn and 5.61% in winter (χ^2^ = 126.29, *p* < 0.001). By 2023, the seasonal pattern had shifted: positivity peaked in spring (24.14%) and summer (29.09%), then decreased in autumn (7.76%) and winter (1.07%) (χ^2^ = 917.55, *p* < 0.001). A similar spring–summer predominance persisted in 2024, with rates of 23.56%, 21.64%, 11.77% and 3.51% in spring, summer, autumn and winter, respectively (χ^2^ = 721.83, *p* < 0.001). Together with the monthly curves in Figure 1, these findings indicate a shift in RSV seasonality on Hainan Island from a summer–autumn peak before 2022 to a spring–summer predominance after the end of the Dynamic Zero-COVID policy (Table 2, Figure 1).

### 3.3. Age-Based Analysis of RSV Infections

RSV-positive patients were categorized into four age groups (0–1, 1–3, 3–7, and 7–18 years) to reflect key developmental stages and typical childcare and schooling transitions in China (infants, toddlers, kindergarten-aged children, and school-aged children and adolescents) [13]. Between 2021 and 2024, the highest RSV prevalence was observed in infants 0–1 years old, with a positivity rate of 22.55% (2228/9882). This was followed by children 1–3 years (17.86%; 1438/8051). RSV prevalence was lower in older age groups, at 7.06% (728/10,314) in children 3–7 years and 2.18% (89/4082) in those 7–18 years (Table 3). Differences among age groups were statistically significant (χ^2^ = 1597.85, *p* < 0.001).

**Figure 2:**
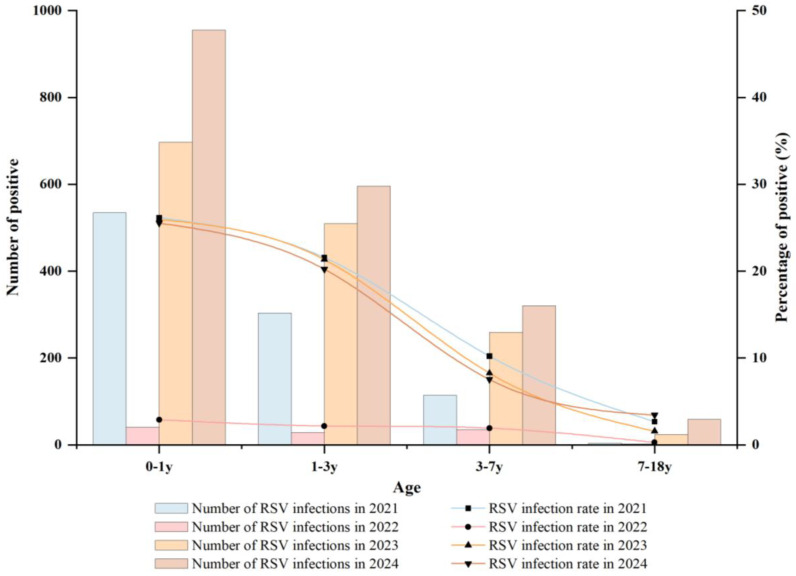
Age distribution and RSV positivity among hospitalized pediatric patients with ARTIs, January 2021–December 2024. RSV-positive cases (bars, left y-axis) and RSV positivity (%) (lines, right y-axis) are stratified by study year (2021–2024) for each age group (0–1, 1–3, 3–7, and 7–18 years).

As shown in Figure 2, age-specific RSV positivity is presented separately for each study year (2021–2024) to facilitate visual comparison of potential post-NPI shifts. As illustrated in Figure 2, RSV infections were concentrated in younger children across all four years. Both the absolute number of RSV-positive cases and the infection rate showed a clear decreasing gradient with increasing age, with the majority of infections occurring in children under 3 years of age. To further quantify potential post-NPI changes in age distribution, we compared the proportions of RSV-positive inpatients aged <1 year and <3 years between 2021 and 2023–2024: the proportion < 1 year decreased from 55.90% (535/957) to 48.30% (1652/3420) (χ^2^ = 17.28, *p* < 0.001), and the proportion < 3 years decreased from 87.67% (839/957) to 80.64% (2758/3420) (χ^2^ = 25.21, *p* < 0.001). We additionally compared age composition across the four predefined strata (0–1, 1–3, 3–7, and 7–18 years) between 2021 and 2023–2024. The proportion of RSV-positive inpatients aged 1–3 years was similar between periods (31.77%, 304/957 vs. 32.34%, 1106/3420; χ^2^ = 0.11, *p* = 0.74), whereas the proportion aged 3–7 years increased (11.91%, 114/957 vs. 16.93%, 579/3420; χ^2^ = 14.13, *p* < 0.001). The proportion aged 7–18 years also differed (0.42%, 4/957 vs. 2.43%, 83/3420; χ^2^ = 15.49, *p* < 0.001), although the number of older cases in 2021 was small. These data indicate that infants and toddlers bear the greatest burden of hospitalized RSV infection in this tropical setting and represent priority groups for targeted prevention and clinical preparedness.

### 3.4. Phylogenetic Analysis of RSV

**Figure 3:**
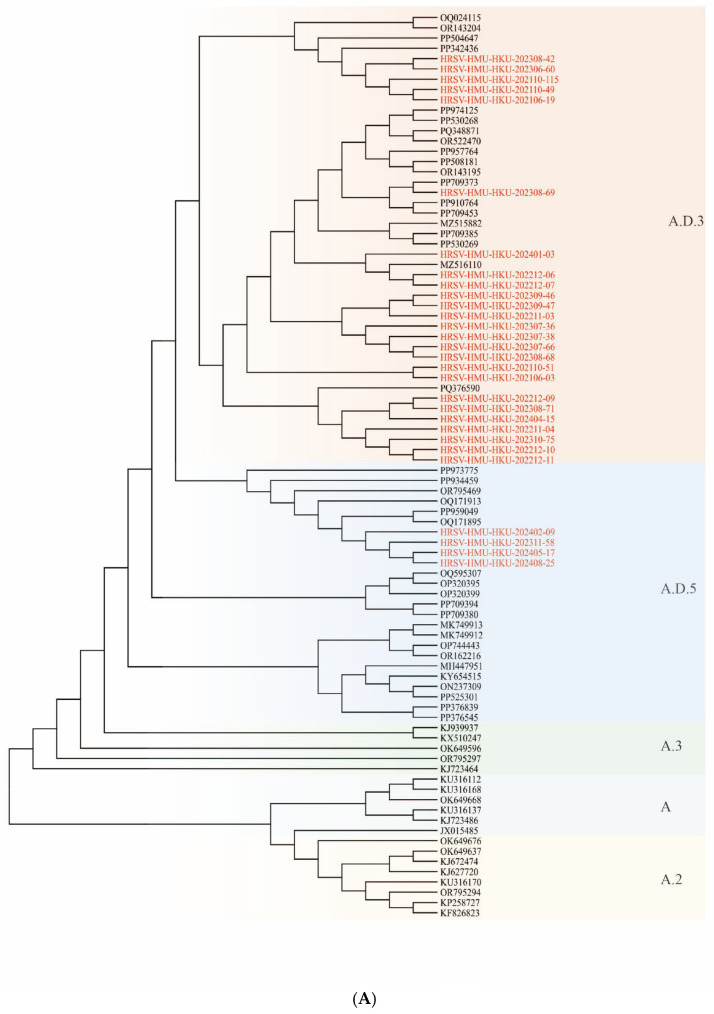

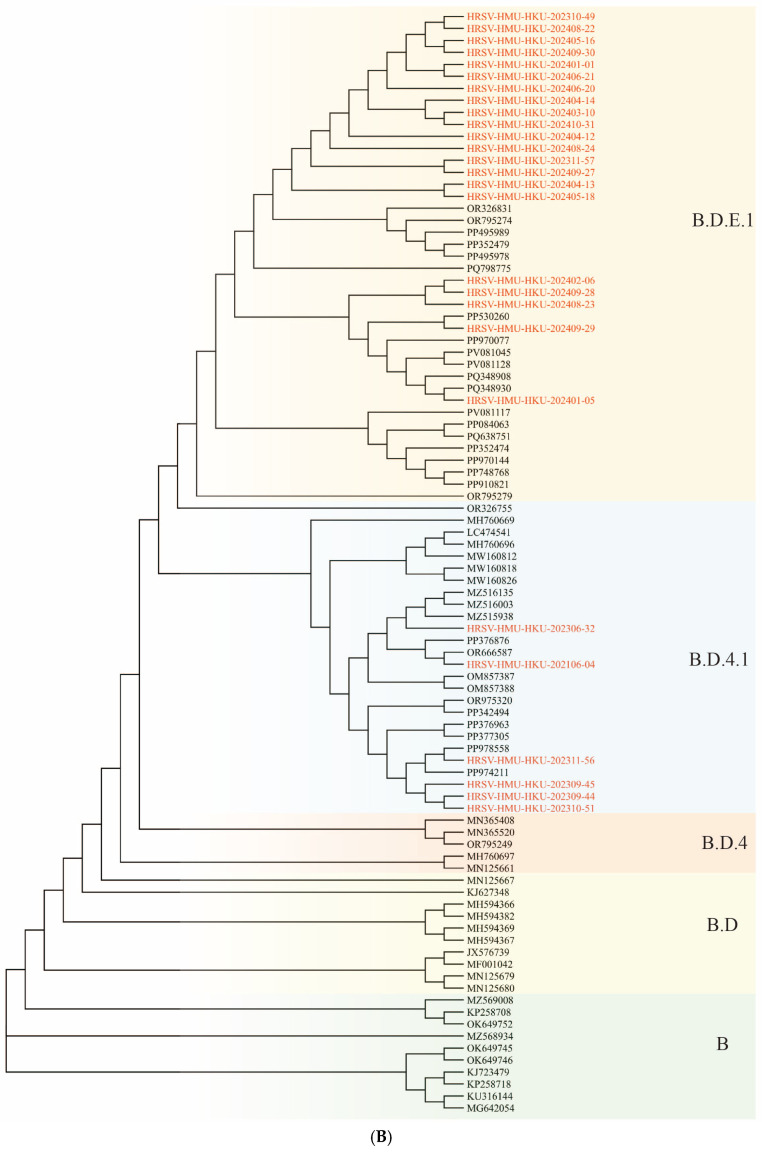
Phylogenetic analysis of RSV strains detected on Hainan Island based on partial G-gene sequences. (**A**) RSV-A; (**B**) RSV-B. Trees were constructed using partial G-gene sequences from this study together with representative reference sequences. Genotypes/lineages are labeled as shown. Hainan sequences are highlighted in the figure. Scale bars indicate the number of substitutions per site.

Partial G-gene sequences were successfully obtained from 56 RSV-positive specimens, including 29 RSV-A and 27 RSV-B strains. All RSV-A sequences belonged to the ON1 genotype and clustered within A.D lineages; specifically, they grouped into A.D.3 and A.D.5.2 (both GA2.3.5-related). All RSV-B sequences belonged to the BA9 genotype and were assigned to B.D.4.1.1, B.D.E.1 or B.D.E.2 lineages (GB5.0.5a-related) (Figure 3A,B).

From 2021 to 2023, RSV-A predominated in Hainan, with an overall RSV-A:RSV-B ratio of approximately 3:1 and A.D.3 representing the most frequently detected lineage. In 2024, a marked shift in subtype predominance was observed: RSV-B became dominant, with an RSV-A:RSV-B ratio of approximately 1:3.8. During the 2021–2023 epidemic seasons, A.D.3 was the main RSV-A lineage, whereas in 2024 A.D.5.2 gradually replaced A.D.3. For RSV-B, only a single B.D.4.1.1 strain was detected in 2021–2022. In 2023, RSV-B sequences were distributed among three lineages, with B.D.4.1.1 being predominant and B.D.E.1 and B.D.E.2 also present. By 2024, all sequenced RSV-B strains belonged to the B.D.E.1 lineage, indicating predominant replacement of RSV-B from B.D.4.1.1 to B.D.E.1 (Figure 3A,B).

To explore potential clinical correlates of RSV type and lineage among sequenced cases, we conducted descriptive comparisons of available clinical variables. Clinical characteristics were first compared between RSV-A and RSV-B infections (Table S2), and then compared between major lineages within each type (RSV-A: A.D.3 vs. A.D.5.2; RSV-B: B.D.4.1.1 vs. B.D.E.1) (Tables S3 and S4). Across the variables available in our dataset—including sex, length of hospital stay, common presenting symptoms, and recorded severity indicators such as severe disease classification and ventilation requirement—no clear differences were observed between the compared groups. These comparisons are descriptive and should be interpreted cautiously due to the limited sample size within some lineages.

### 3.5. Amino Acid Analysis

**Figure 4:**
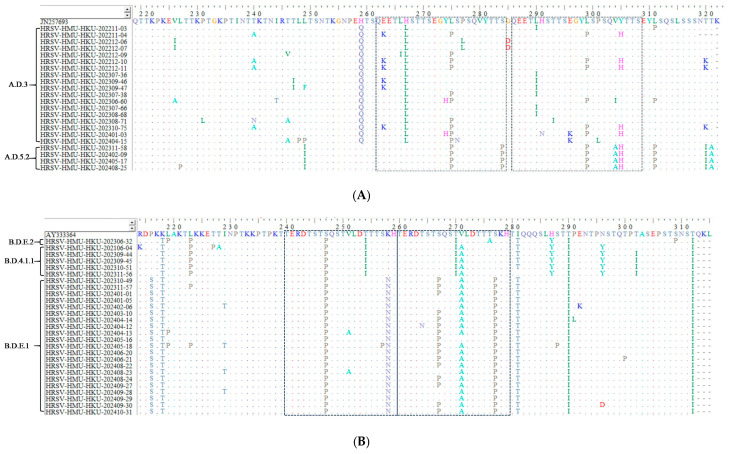
Amino-acid alignments of the second hypervariable region (HVR2) of the RSV G protein. (**A**) RSV-A strains aligned to the ON1 prototype strain (GenBank: JN257693); alignment corresponds to amino-acid positions 227–321 of the G protein. (**B**) RSV-B strains aligned to the BA9 prototype strain (GenBank: AY333364); alignment corresponds to amino-acid positions 214–315. Identical residues are indicated by dots. The duplicated segments are marked by dotted boxes (23 amino acids for RSV-A and 20 amino acids for RSV-B).

To characterize lineage-specific amino-acid changes, we compared the deduced G-protein sequences of RSV strains from Hainan with prototype ON1 (JN257693) and BA9 (AY333364) strains. The analyzed regions encompassed the second hypervariable region (HVR2), including the 23-amino-acid duplication in RSV-A and the 20-amino-acid duplication in RSV-B (Figure 4A,B).

Among RSV-A strains, all A.D.3 sequences shared H258Q and H266L substitutions. Other frequent substitutions included L274P (10/18), L289I (7/18), L298P (6/18), E262K (6/18), Y305H (5/18), T239A (4/18) and T319K (3/18). In contrast, all A.D.5.2 strains exhibited a highly conserved pattern of concurrent mutations at L248I, L274P, S283P, L298P, V303A, Y304H, L310I and T320A (Figure 4A).

Predicted N-linked glycosylation also differed by RSV-A lineage. Relative to the ON1 prototype, nearly all A.D.3 strains were predicted to harbor five N-glycosylation sites (N85, N103, N135, N179 and N237). To avoid ambiguity in residue numbering, all site positions are reported relative to the ON1 prototype sequence (JN257693), and predicted N-linked glycosylation is interpreted based on the canonical consensus sequon N–X–S/T (X ≠ Pro). In our dataset, the A.D.3-associated “N179” locus corresponds to the presence of an N179–X180–S/T181 tripeptide that satisfies this consensus, whereas the corresponding tripeptide in the ON1 prototype and in most A.D.5.2 sequences does not satisfy the N–X–S/T rule and therefore is not predicted as glycosylated at this locus. In addition, an S100N substitution detected in five strains collected between July and August 2023 introduced an extra predicted N-glycosylation site at N100. At the motif level, this change converts the local tripeptide at residues 100–102 from S100–X101–S/T102 to N100–X101–S/T102, thereby creating an N–X–S/T sequon. In contrast, almost all A.D.5.2 strains retained four N-glycosylation sites (N85, N103, N135 and N237) but lacked the N179 site observed in A.D.3 strains (Table 4). Because these glycosylation sites are computational predictions, they should be interpreted cautiously and ideally validated by functional assays.

For RSV-B, all B.D.4.1.1 strains carried a shared set of substitutions—K218T, L223P, S247P, T254I, T270I, V271A, H287Y, T290I, N296Y and T312I—with additional frequent changes at I286T (4/5, 82%) and T302I (4/5, 82%) (Figure 4B). All B.D.E.1 strains showed a distinct, relatively uniform mutation profile involving P216S, K218T, S247P, K258N, S277P, I281T, T290I and T312I, with common additional substitutions at V271A (20/21), S267P (16/21) and I229T (4/21).

Predicted N-glycosylation patterns of RSV-B also differed by lineage. B.D.4.1.1 strains had a single N-glycosylation site at N230; the combination of N296Y and T312I abolished predicted glycosylation sites in the C-terminal region of the G protein. In contrast, B.D.E.1 strains were predicted to contain three N-linked glycosylation sites at N230, N258 and N296. The K258N substitution, present in all B.D.E.1 sequences, created a novel N258 site. None of the B.D.E.1 strains retained the N310 site, likely due to T312I disrupting the N-X-S/T consensus motif required for N-linked glycosylation (Table 5). Consistent with the N–X–S/T rule, the T312I substitution disrupts the local sequon at residues 310–312 by converting an N310–X311–T312 motif into N310–X311–I312, thereby abolishing the predicted N310 site.

## 4. Discussion

This four-year study from tropical Hainan Island shows that RSV epidemiology and viral population structure in hospitalized children were profoundly reshaped across the implementation and withdrawal of China’s Dynamic Zero-COVID policy. RSV activity was markedly suppressed in 2022, then rapidly re-established in 2023–2024 to near-pre-pandemic levels, accompanied by an advance of epidemic timing from a summer–autumn peak to spring–summer predominance and by clear lineage turnover within both RSV-A and RSV-B. Together, these findings highlight how non-pharmaceutical interventions (NPIs), population immunity, and viral evolution jointly determine RSV transmission dynamics, even in a tropical climate.

The abrupt reduction in RSV positivity in 2022 is temporally consistent with intensive NPIs implemented during two local COVID-19 waves, supporting the interpretation that decreased contact rates and mobility can interrupt RSV circulation. Importantly, NPI intensity and local epidemic pressure were not uniform across the pandemic period; despite broader COVID-19 control policies in 2021, Hainan experienced comparatively limited local outbreaks and more sustained reopening of schools and population mobility than during the two major waves in 2022, which may have allowed RSV transmission to persist and contributed to the higher hospital-based positivity observed in 2021. In Hainan, these NPIs were implemented during two major local COVID-19 waves (approximately March–April and August–September 2022) and included intensified testing and isolation, targeted movement restrictions, and short-term suspensions of schools and kindergartens followed by staged reopening; importantly, these measures also perturbed tourism-related population flows, which are a key feature of this tropical island setting. Following nationwide relaxation of NPIs in late 2022, RSV rebounded quickly and peaked earlier in the year. Similar post-NPI “off-season” or advanced RSV epidemics have been widely documented, and are most commonly explained by (i) immunity debt, whereby reduced exposure during NPI periods increases the pool of susceptible infants and toddlers, and (ii) ecological interactions among respiratory viruses, including both interference (e.g., interferon-mediated) and facilitation related to co-circulation and altered host susceptibility [17,18]. Consistent with this global experience, large interseasonal or out-of-season RSV resurgences were reported after easing of COVID-19 restrictions in Australia, including marked summer outbreaks in 2020–2021, and in the United States, where RSV seasonality shifted during 2021–2023 compared with pre-pandemic patterns [19]. Comparable disruptions were also described in Europe, including year-round or interseasonal transmission following a summer 2021 outbreak in the Netherlands and an interseasonal resurgence associated with changing public health measures in France, as well as in Japan where unusually high RSV activity was reported after resumption of social activities for children [20,21,22]. To provide additional ecological context, we summarized co-detections within the 56 RSV-positive specimens selected for G-gene sequencing: RSV mono-infection accounted for 19/56 cases, whereas 37/56 had one or more co-detected pathogens (Tables S5 and S6). This co-detection summary is descriptive and limited to the sequenced subset, and we did not perform cohort-wide comparisons between RSV-positive and RSV-negative patients or infer causal interference from retrospective co-detection data. Consistent with this framework, our contemporaneous surveillance in Hainan suggested broader ecological re-organization of pediatric respiratory viruses during the same period, with altered seasonal patterns and rebound dynamics as NPIs were relaxed. Taken together, the Hainan-specific disruption of contact patterns and population mobility in 2022, coupled with accumulated susceptibility and virus–virus interactions, provides a coherent explanation for the sharp RSV suppression in 2022 and the subsequent resurgence with shifted seasonality in 2023–2024.

This pattern is also consistent with broader respiratory virus re-organization in Hainan observed in our team’s prior studies: influenza virus, rhinovirus and Human parainfluenza virus showed pronounced NPI-related perturbations and altered seasonal timing after relaxation, whereas rhinovirus circulation was comparatively resilient [12,13,14]. Such differences underscore virus-specific sensitivity to behavioural interventions and the likelihood that RSV resurgence reflects both restored contact networks and a widened susceptible population.

Despite major temporal shifts, the demographic signature of RSV in Hainan remained typical. Infants < 1 year carried the highest burden, followed by children 1–3 years, with a steep decline in older age groups. This age gradient aligns with global evidence that RSV hospitalization burden is concentrated in the first two years of life, plausibly driven by immature immunity, smaller airway caliber, and limited prior exposure [2,23].

RSV positivity was consistently higher in boys than girls in most years. Male predominance has been repeatedly reported in RSV cohorts and likely reflects a combination of biological susceptibility and behavioural or care-seeking factors [24,25]. These stable demographic patterns, observed across periods of suppression and resurgence, reinforce the need to prioritize very young children—particularly infants—for prevention strategies as RSV immunization tools become more widely implemented.

All sequenced RSV-A strains belonged to genotype ON1 and all RSV-B strains to BA9, matching their long-standing global predominance [25,26]. Within these genotypes, however, we observed clear lineage replacement. RSV-A predominated during 2021–2023, mainly as A.D.3, while RSV-B became dominant in 2024, driven almost entirely by B.D.E.1. Concurrently, A.D.5.2 increased and replaced A.D.3 as the major RSV-A lineage by 2024, and B.D.E.1 replaced B.D.4.1.1 within RSV-B. This sequence of events is consistent with recent reports from other parts of China indicating that A.D.5.2 and B.D.E.1 expanded rapidly after relaxation of travel and control measures [11,27].

Mechanistically, several non-exclusive processes may explain the observed turnover: founder effects linked to re-introduction after prolonged suppression; altered immune landscapes created by “gaps” in exposure during NPIs; and lineage-specific fitness advantages related to transmissibility, immune escape, or attachment [13,28]. RSV evolution is characterized by strong purifying selection across most of the genome but repeated adaptive change in surface glycoproteins—particularly G—making lineage replacement a plausible outcome when susceptible pools expand and introductions resume.

Lineage-specific amino-acid substitutions and predicted N-glycosylation patterns in the G protein provide a molecular correlate to these epidemiological shifts. In RSV-A, A.D.3 strains shared conserved H258Q and H266L substitutions plus additional variable changes (e.g., E262K, L274P, L298P, Y305H), consistent with ongoing intra-lineage diversification [4,29]. A.D.5.2 strains exhibited a more stereotyped substitution set (L248I, L274P, S283P, L298P, V303A, Y304H, L310I, T320A), suggesting a comparatively conserved profile. Predicted glycosylation differed meaningfully between these RSV-A lineages. Most A.D.3 strains carried N-linked sites at N85, N103, N135, N179, and N237, with a subset gaining N100 (consistent with S100N). In contrast, A.D.5.2 strains lacked N179 while retaining N85, N103, N135, and N237. Because N179 lies within the cysteine-noose region embedded in broadly neutralizing epitope landscapes, its absence in A.D.5.2 could alter epitope accessibility and antibody recognition [30,31]. Although our analysis is predictive, these patterns support the hypothesis that A.D.5.2 may differ antigenically from A.D.3 in ways that could influence population-level susceptibility.

In RSV-B, B.D.4.1.1 strains carried a characteristic panel of substitutions (including N296Y and T312I) and were predicted to retain N230 as the dominant C-terminal glycosylation site, consistent with loss of other C-terminal sites [21,32,33]. The emergent B.D.E.1 lineage—predominant in 2024—was characterized by changes including P216S and K258N, with predicted glycosylation typically at N230, N258, and N296. K258N introduces a novel N258 site, while T312I disrupts the N310 site. We also observed minor heterogeneity within B.D.E.1 (a small subset lacking N296 and/or N258), indicating ongoing fine-scale diversification even within a rapidly expanding lineage.

Collectively, these substitutions and glycosylation changes imply re-configuration of the G-protein glycan shield, a plausible mechanism for immune evasion and improved fitness in partially immune populations [18]. Given this potential functional relevance, we further examined whether major lineages differed in clinical presentation among the sequenced cases. As summarized in Tables S2–S4, we did not identify consistent differences in length of hospitalization, common symptoms, or the proportions classified as severe or requiring ventilation when comparing RSV-A versus RSV-B or comparing dominant lineages within each type (A.D.3 vs. A.D.5.2; B.D.4.1.1 vs. B.D.E.1). These comparisons should be interpreted as exploratory because the number of sequenced specimens within some lineages was limited and statistical power was modest. Functional studies—confirming glycosylation, mapping antigenicity, and assessing binding/neutralization—will be needed to establish causal links between these molecular signatures and potential transmission advantages.

The spring–summer predominance observed in 2023–2024 suggests that the optimal timing of future RSV prophylaxis on Hainan may differ from pre-pandemic patterns and from schedules derived in temperate climates. As long-acting monoclonal antibodies and maternal vaccination strategies become available, locally calibrated seasonality data will be important to maximize effectiveness. For example, a practical approach for nirsevimab-like prophylaxis would be to prioritize administration shortly before the anticipated spring–summer surge, so that protection overlaps with the local high-transmission period. Similarly, maternal RSV vaccination could be timed in late pregnancy in the months preceding the spring–summer peak to maximize transplacental antibody protection for newborns entering the seasonal rise. Although current leading interventions primarily target the prefusion F protein (rather than G), continued genomic surveillance remains essential. G-protein evolution can shape transmissibility and antigenic context, and expanding immunization may introduce new selection pressures that are best captured by integrated whole-genome surveillance and linkage to clinical outcomes.

This study has several limitations. It was conducted at a single tertiary center and included only hospitalized children, potentially under-representing mild/community infections and biasing toward severe disease. In addition, comparable routine RSV surveillance using the same clinical testing workflow was not available at our center in 2018–2020, which limits direct pre-pandemic baseline comparisons of RSV magnitude and seasonality and constrains inference about how unusual the 2021 patterns were relative to earlier years. RSV positivity was defined based on the final qualitative call from a commercial targeted NGS respiratory panel used for routine clinical testing; although QC-passed results were used throughout, commercial assays may undergo internal updates (e.g., panel content, sequencing instruments, reference databases, or bioinformatics interpretation rules) that are not fully transparent to end users, which could introduce unmeasured variability over time. The number of sequenced specimens was limited, and analysis focused on partial G-gene regions rather than whole genomes, restricting resolution of genome-wide evolutionary dynamics and limiting assessment of genotype–severity associations. In addition, N-linked glycosylation inferences were limited to predicted motifs within the sequenced HVR2 fragment and may not capture potential N-linked sites elsewhere in the full-length G protein. Although we performed descriptive comparisons of available clinical variables by RSV type and lineage (Tables S2–S4), ICU admission and other ICU-level severity metrics were not systematically captured or could not be consistently linked to all sequenced specimens in this retrospective dataset, and statistical power for lineage-specific clinical comparisons was limited. Moreover, co-detection analyses were summarized only for the 56 sequenced RSV-positive specimens (Tables S5 and S6) rather than the full RSV-positive and RSV-negative cohort; therefore, we could not robustly compare co-detection patterns between RSV-positive and RSV-negative patients, nor infer causal viral interference from these retrospective co-detection data. Future work should expand multicenter sampling across Hainan, include outpatient/community cohorts, increase sequencing density with whole-genome approaches, and integrate clinical severity metrics, co-infection data, and (ideally) longitudinal serology to disentangle the contributions of immunity debt, viral interactions, and viral evolution.

In summary, RSV circulation on tropical Hainan rebounded rapidly after NPI relaxation with earlier seasonal peaks, a switch from RSV-A to RSV-B predominance, and lineage replacement within ON1 and BA9 genotypes. Distinct lineage-specific amino-acid and glycosylation signatures in the G protein suggest ongoing antigenic remodeling, underscoring the value of sustained genomic and epidemiological surveillance to inform timing and targeting of RSV immunization strategies in tropical regions.

## Figures and Tables

**Table 1:** RSV prevalence among hospitalized pediatric patients with ARTIs by sex, 2021–2024.

Gender	2021	2022	2023	2024	Total
Male	618/3002 (20.57)	54/3173 (1.70)	968/5951 (16.27)	1192/7507 (15.88)	2832/19,633 (14.42)
Female	339/1719 (19.72)	52/2054 (2.53)	522/3781 (13.81)	738/5142 (14.35)	1651/12,696 (13.00)
χ^2^ value	0.51	4.32	10.79	5.50	13.03
*p* value	0.477	0.038	0.001	0.019	<0.001

**Table 2:** RSV prevalence among hospitalized pediatric patients with ARTIs by season, 2021–2024.

Season	2021	2022	2023	2024
Spring	56 (5.85)	16 (0.93)	569 (24.14)	926 (23.56)
Summer	422 (37.54)	1 (0.07)	655 (29.09)	632 (21.64)
Autumn	462 (29.20)	12 (1.57)	245 (7.76)	240 (11.77)
Winter	17 (1.61)	77 (5.61)	21 (1.07)	132 (3.51)
χ^2^ value	636.76	126.29	917.55	721.83
*p* value	<0.001	<0.001	<0.001	<0.001

**Table 3:** RSV prevalence among hospitalized pediatric patients with ARTIs by age, 2021–2024.

Age	2021	2022	2023	2024	Total	χ^2^ Value	*p* Value
0–1 y	535/2045 (26.16)	41/1413 (2.90)	697/2684 (25.97)	955/3740 (25.53)	2228/9882 (22.55)	364.70	<0.001
1–3 y	304/1409 (21.57)	28/1305 (2.15)	510/2391 (21.33)	596/2946 (20.23)	1438/8051 (17.86)	263.83	<0.001
3–7 y	114/1117 (10.20)	35/1809 (1.93)	259/3140 (8.25)	320/4248 (7.53)	728/10,314 (7.06)	97.50	<0.001
7–18 y	4/150 (2.67)	2/700 (0.29)	24/1517 (1.58)	59/1715 (3.44)	89/4082 (2.18)	27.26	<0.001
Total	957/4721 (20.27)	106/5227 (2.03)	1490/9732 (15.31)	1930/12,649 (15.26)	4483/32,329 (13.86)	812.97	<0.001
χ^2^ value	144.17	16.293	643.24	743.12	1597.85	/	
*p* value	<0.001	<0.001	<0.001	<0.001	<0.001		

**Table 4:** Prediction of N-glycosylation sites in the G gene of RSV-A strains found in Hainan Province.

Lineage	Sample Number	N85	N100	N103	N135	N179	N237
ON1							
	JN257693	+		+	+		+
A.D.3							
	HRSV-HMU-HKU-202211-03	+		+	+	+	+
	HRSV-HMU-HKU-202211-04	+		+	+	+	
	HRSV-HMU-HKU-202212-06	+		+	+	+	+
	HRSV-HMU-HKU-202212-07	+		+	+	+	+
	HRSV-HMU-HKU-202212-09	+		+	+	+	+
	HRSV-HMU-HKU-202212-10	+		+	+	+	
	HRSV-HMU-HKU-202212-11	+		+		+	
	HRSV-HMU-HKU-202401-03	+		+	+	+	+
	HRSV-HMU-HKU-202404-15	+		+	+	+	+
	HRSV-HMU-HKU-202307-36	+	+	+	+	+	+
	HRSV-HMU-HKU-202309-46	+		+	+	+	+
	HRSV-HMU-HKU-202309-47	+		+	+	+	+
	HRSV-HMU-HKU-202307-38	+	+	+	+	+	+
	HRSV-HMU-HKU-202306-60	+		+	+	+	+
	HRSV-HMU-HKU-202307-66	+	+	+	+	+	+
	HRSV-HMU-HKU-202308-68	+	+	+	+	+	+
	HRSV-HMU-HKU-202308-71	+	+	+	+	+	
	HRSV-HMU-HKU-202310-75	+		+	+	+	
A.D.5.2							
	HRSV-HMU-HKU-202408-25	+		+	+		+
	HRSV-HMU-HKU-202405-17	+		+	+		+
	HRSV-HMU-HKU-202311-58	+		+	+		+
	HRSV-HMU-HKU-202402-09	+		+	+		+

**Table 5:** Prediction of N-glycosylation sites in the G gene of RSV-B strains found in Hainan Province.

Lineage	Sample Number	N230	N258	N296	N310
BA9					
	AY333364	+		+	+
B.D.4.1.1					
	HRSV-HMU-HKU-202306-32	+		+	
	HRSV-HMU-HKU-202106-04	+			
	HRSV-HMU-HKU-202309-44	+			
	HRSV-HMU-HKU-202309-45	+			
	HRSV-HMU-HKU-202310-51	+			
B.D.E.1					
	HRSV-HMU-HKU-202311-56	+			
	HRSV-HMU-HKU-202310-49	+	+	+	
	HRSV-HMU-HKU-202311-57	+	+	+	
	HRSV-HMU-HKU-202401-01	+	+	+	
	HRSV-HMU-HKU-202401-05	+	+	+	
	HRSV-HMU-HKU-202402-06	+	+	+	
	HRSV-HMU-HKU-202403-10	+	+	+	
	HRSV-HMU-HKU-202404-14	+	+	+	
	HRSV-HMU-HKU-202404-12	+	+	+	
	HRSV-HMU-HKU-202404-13	+	+	+	
	HRSV-HMU-HKU-202405-16	+	+	+	
	HRSV-HMU-HKU-202405-18	+	+	+	
	HRSV-HMU-HKU-202406-20	+	+	+	
	HRSV-HMU-HKU-202406-21	+	+	+	
	HRSV-HMU-HKU-202408-22	+	+	+	
	HRSV-HMU-HKU-202408-23	+	+	+	
	HRSV-HMU-HKU-202408-24	+	+	+	
	HRSV-HMU-HKU-202409-27	+	+	+	
	HRSV-HMU-HKU-202409-28	+	+	+	
	HRSV-HMU-HKU-202409-29	+	+	+	
	HRSV-HMU-HKU-202409-30	+	+		
	HRSV-HMU-HKU-202410-31	+	+	+	

## Data Availability

All sequences analyzed during this study are available from the NCBI database (GenBank accession No. OQ248592-OQ248607, OR140542-OR140554 and PV800154-PV800198).

## Supplementary Materials

The following supporting information can be downloaded at: https://www.mdpi.com/article/10.3390/pathogens15020182/s1, Table S1: Comparison of demographic and temporal distribution characteristics between sequenced RSV-positive cases and all RSV-positive cases; Table S2: Clinical characteristics of patients with different genotypes and disease severity; Table S3: Clinical characteristics of patients with different RSV-A genotypes and disease severity; Table S4: Clinical characteristics of patients with different RSV-B genotypes and disease severity; Table S5: The frequency of RSV infection alone and mixed infections with different numbers of viruses; Table S6: The frequency of mixed viral infections in RSV patients.
