# Supplementary material for: Molecular Lineage Replacement and Shifted Seasonality of Pediatric Respiratory Syncytial Virus on Tropical Hainan Island, China, 2021–2024
Source: Pathogens. 2026 Feb 6;15(2):182. doi: 10.3390/pathogens15020182 (PMC12943523; doi:10.3390/pathogens15020182)
Supplement: Supplementary file 1 [file pathogens-15-00182-s001.zip › pathogens-4089246-supplementary.pdf]

Table S1. Comparison of demographic and temporal distribution characteristics between sequenced RSV-positive cases and all RSV-positive cases.

Variable	Sequenced RSV-positive (n=56)	Total cases RSV-positive (n=4,483)	P value
**Age, years, mean ± SD**	2.05 ± 1.84	1.95 ± 2.38	0.69
**Age group, n (%)**			
0–1	30（53.57）	2228（49.70）	0.74
1–3	15（26.79）	1438（32.08）	
3–7	9（16.07）	728（16.24）	
7–18	2（3.57）	89（1.99）	
**Sex, n (%)**			
Male	32（57.14）	2832（63.17）	0.35
Female	24（42.86）	1651（36.83）	
**Year of detection, n (%)**			
2021	9（10.71）	957（21.35）	0.24
2022	4（12.50）	106（2.36）	
2023	19（33.93）	1490（33.24）	
2024	24（42.86）	1930（43.05）	

Table S2.Clinical characteristics of patients with different genotypes and disease severity

Variable	RSV-A(n = 29)	RSV-B (n = 27)	*p*-value
Male/female	16/13	16/11	0.76
Hospital stays, days	6.41 ± 3.53	6.44 ± 5.32	0.98
Cough	27	25	1
Fever	21	17	0.23
Nasal congestion	10	10	0.84
Cough up phlegm	22	18	0.45
Wheeze	12	0	＜0.00
Sneeze	1	0	1
Respiratory failure	0	1	0.97
Tachypnea	0	2	0.44
Severe	3	4	0.83
Ventilation requirement	2	3	0.93

Table S3.Clinical characteristics of patients with different RSV-A genotypes and disease severity

**Variable**	**A.D.5(n = 4)**	**A.D.3(n = 25)**	***p*-value**
Cough	4	23	1
Fever	1	20	0.92
Nasal congestion	3	7	0.20
Cough up phlegm	3	19	1
Wheeze	3	9	0.36
Sneeze	0	1	1
Respiratory failure	0	0	/
Tachypnea	0	0	/
Severe	1	2	0.88
Ventilation requirement	0	2	1

Table S4.Clinical characteristics of patients with different RSV-B genotypes and disease severity

**Variable**	**B.D.4.1(n = 6)**	**B.D.E.1 (n = 21)**	***p*-value**
Cough	6	19	1
Fever	3	14	0.79
Nasal congestion	2	8	1
Cough up phlegm	3	16	0.46
Wheeze	0	0	/
Sneeze	0	0	/
Respiratory failure	0	1	1
Tachypnea	0	2	1
Severe	2	2	0.43
Ventilation requirement	2	1	0.22

Table S5.The frequency of RSV infection alone and mixed infections with different numbers of viruses

Infection type	Number of cases
RSV	19
Co-infection with 1 pathogen	16
Co-infection with 2 pathogens	12
Co-infection with 3 pathogens	8
Co-infection with 4 pathogens	1

Table S6.The frequency of mixed viral infections in RSV patients

Pathogen name	Number of mixed infections
Streptococcus pneumoniae	12
Cytomegalovirus	9
Haemophilus influenzae	9
Moraxella catarrhalis	9
Staphylococcus aureus	8
Enterovirus	5
Rhinovirus	4
